# Association of knee joint performance and gait patterns with pain catastrophizing in patients with severe knee osteoarthritis: a cross-sectional study

**DOI:** 10.1186/s12891-025-08993-2

**Published:** 2025-07-28

**Authors:** Hideaki Matsuo, Masafumi Kubota, Hiroaki Naruse, Kazuki Shoji, Daiki Hasegawa, Yudai Watabe, Takumi Sakamoto, Akihiko Matsumine

**Affiliations:** 1 Division of Physical Therapy and Rehabilitation Medicine, University of Fukui Hospital, 23-3 Matsuoka Shimoaizuki, Eiheiji-cho, Fukui 910-1193 Japan; 2 Faculty of Health Sciences, Institute of Medical, Pharmaceutical and Health Sciences, Kanazawa University, Kanazawa, Japan; 3 Department of Orthopaedics and Rehabilitation Medicine, Faculty of Medical Sciences, University of Fukui, Matsuoka Shimoaizuki 23, Eiheiji, Fukui 910-1193 Japan

**Keywords:** Pain catastrophizing, Knee osteoarthritis, Range of motion, Muscle strength, Knee biomechanics

## Abstract

**Background:**

Pain catastrophizing is linked to patient-reported outcomes in knee osteoarthritis (OA) patients, but its correlation with objective joint-level function (range of motion and strength) and gait patterns is unclear. This study examined the association between objective knee function, gait patterns, and pain catastrophizing in severe knee OA patients.

**Methods:**

This cross-sectional study included patients with knee OA admitted for total knee arthroplasty over a 3-year period from November 2016. Medical records and instrumentation databases were assessed. Patients who completed the Pain Catastrophizing Scale (PCS) were categorized into high PCS (scores ≥ 30) and low PCS (scores

**Results:**

The analysis included 63 patients with 75 knees divided into 60 knees in the high-PCS group and 15 knees in the low-PCS group. No significant differences were found in objective knee function and gait parameters between the groups. Regression analysis showed no association between PCS and gait variables or objective knee function.

**Conclusions:**

No significant associations were observed between pain catastrophizing and objective knee function (range of motion and muscle strength) or knee biomechanics during gait in patients with severe knee OA. These findings suggest that these aspects may be independently evaluated in the clinical assessment of disability in this population.

**Supplementary Information:**

The online version contains supplementary material available at 10.1186/s12891-025-08993-2.

## Introduction

Knee osteoarthritis (OA) is the most common disease, with pain as the primary symptom, with a reported prevalence of 47.0% in men and 70.2% in women among Japanese aged ≥ 60 years [1, 2]. Severe knee OA is a major factor that adversely affects the daily lives of elderly individuals [3, 4]. In recent years, because radiographic findings and knee biomechanics do not always correspond to pain intensity in patients with knee OA [5, 6], pain phenotypes defined by various characteristics have attracted attention [7]. The determination of the relationships between symptoms and findings is necessary to develop individualized treatment based on patient characteristics.

Pain catastrophizing is defined as an exaggerated negative mindset brought to bear during an actual or anticipated pain experience [8]. It is characterized by the tendency to magnify the threat value of pain stimulus, to feel helpless in the context of pain, and by a relative inability to inhibit pain-related thoughts in anticipation of, during, or following a painful encounter [9]. In patients with knee OA, pain catastrophizing is a factor that defines the pain phenotype [7] and has been reported as a multiple subjective preoperative predictor of postoperative outcome [10–12]. In addition, therapeutic interventions for pain catastrophizing are partially effective [13]. Although the relationship between catastrophic thoughts and questionnaire-assessed knee function has been reported in patients with knee OA [14], the relationship between pain catastrophizing and objective knee function, such as joint range of motion (ROM) and muscle strength, is yet to be fully clarified.

Psychological tendencies, such as pain catastrophizing, which negatively perceive pain, are expected to affect the gait patterns of patients with knee OA. Gait patterns may influence mood in daily life [15]. A previous study examining the effect of pain catastrophizing on gait patterns in patients with severe knee OA reported that participants with higher pain-catastrophizing scores had a smaller mid-stance knee-extension limitation [16]. However, interpretation of this gait pattern as a kinematic characteristic reflecting a psychological state, such as pain catastrophizing, that tends to be perceived pain negatively, is difficult. Thus, it remains unclear whether the gait patterns of patients with knee OA are affected by pain catastrophizing.

As mentioned above, evaluation of pain catastrophizing based on its relationship with objective knee function—specifically, joint-level physical performance such as knee range of motion and muscle strength—is recommended. However, reports investigating the relationship between pain catastrophizing and measurement-based gait patterns or objective knee function are limited. Clarifying the relationship between pain catastrophizing and gait patterns, objective knee function will help clinicians better understand the clinical aspects of pain catastrophizing in patients with severe knee OA. This study aimed to examine the association of objective knee function and gait pattern with pain catastrophizing in patients with severe knee OA. Based on clinical experience, we hypothesized that patients with severe knee OA who exhibit pain catastrophizing will have a gait characterized by avoidance of loading and immobility of the knee joint with a concomitant decrease in objective knee function.

## Materials and methods

### Participants

The subjects were patients diagnosed with knee OA of severity grade 3 or higher on the Kellgren-Lawrence (KL) grade between November 2016 and 3 years thereafter. All the patients were scheduled to undergo knee arthroplasty at our university hospital. Among these patients, those who had difficulty walking 30 m without a walking aid were excluded because it was difficult to perform a gait-analysis test. Patients with rheumatoid arthritis, osteonecrosis, and post-traumatic arthritis were also excluded from the study. Preoperative information was collected and examined retrospectively from the medical records and the database of the three-dimensional motion system. During the study period, knee arthroplasty procedures were performed unilaterally, necessitating that all subsequent evaluations be performed separately on each knee in cases of bilateral surgery. As this was a retrospective, cross-sectional study using existing data, the purpose of the study was explained through the website, and the participants were free to opt out of participation.

### Measurements

For the participants who met the above criteria, we extracted [1] demographic data [2], Pain Catastrophizing Scale (PCS) measurement [3], pain intensity [4], objective knee function (ROM and muscle strength), and [5] gait-analysis parameters.

### Demographic data

The data on age, sex, and affected side were extracted from medical records. Height and weight were measured with a measuring device while the participants were clothed and barefoot. Body mass index (BMI) was calculated by dividing the weight (kg) by height (m) squared.

### Pain catastrophizing

Pain catastrophizing was assessed using the Japanese version of the PCS [17], a self-reported questionnaire that assesses cognitive and emotional responses to pain; the PCS consists of 13 items, each rated on a 5-point Likert scale ranging from 0 (‘not at all’) to 4 (‘all the time’). The total score was calculated by summing all individual item scores (range, 0–52). The PCS covers three dimensions of catastrophizing: rumination, magnification, and helplessness [18]. The higher the score, the higher the presence of catastrophizing thoughts. PCS has high reliability and validity [18, 19], including those for the Japanese version [17].

### Pain intensity

Pain intensity was assessed using a visual analog scale (VAS) for resting and walking. VAS consisted of a 100-mm line on which the participant places a mark between the left side, “0”, representing “no pain,” and the right side, “100”, representing “worst pain imaginable.”

### Objective knee function

Knee joint ROM was measured passively in the supine position using standard goniometry techniques for flexion and extension. The axis of the goniometer was aligned with the center of the knee, fixed arm was aligned with the greater trochanter and lateral femoral condyle, and mobile arm was aligned with the fibular head and lateral ankle. Negative values for knee-extension ROM indicate that the knee joint was not fully extended.

Muscle strength was measured using isokinetic muscle testing of the quadriceps and hamstrings. Measurements were performed using an isokinetic dynamometer (Biodex System 4; Biodex Medical Systems, Shirley, NY, USA) and the procedures described in previous reports [20]. Briefly, the patient was fixed to the system chair and the feet of the patient were attached to the lever arms of the dynamometer. The rotation axis of the dynamometer was visually aligned with the lateral femoral condyle, and the lever arm was set to move at an angular velocity of 60°/s from 90° to 0° of knee flexion. Gravity compensation was performed according to the instructions. Each participant was required to extend and flex the knee “as fast and as hard as possible” three times throughout the entire ROM after performing three familiarization movements at 40–80% of maximum muscle strength. The data on the peak torques of the three trials obtained during a constant angular velocity period were extracted, corrected for body weight, and used as representative values.

### Three-dimensional gait analysis

Three-dimensional gait analysis was performed according to the equipment and procedures described in our previous report [21]. Briefly, we used a three-dimensional motion-analysis system (VICON-MX; Vicon Motion Systems, Oxford, UK) that included ten T10 cameras (Vicon Motion Systems) and four synchronized force plates (AMTI, Watertown, MA, USA) placed in the middle of a 10-m walkway. All cameras and force plates were calibrated prior to data collection. The sampling rate was 100 Hz for the cameras and 1,000 Hz for the force plates. Sixteen retro-reflective markers (14 mm) were attached to each patient on anatomical landmarks following the Plug-in-Gait marker set. The participants were instructed to walk barefoot on a 10-m walkway at a comfortable speed. All participants conducted warm-up trials to habituate them to the gait laboratory setting before the measurement. The measurement ended when five correct trials were recorded.

The trials were visually confirmed to be measured correctly using Vicon NEXUS motion-capture software (Vicon Motion Systems Ltd., Oxford, UK). The marker’s trajectory was reconstructed and filtered using the Woltring filtering routine with Vicon NEXUS. Gait events (heel strikes and toe-offs) were automatically defined using the detect-gait-cycle-event pipeline based on the force-plate data, with a vertical ground reaction force threshold of 20 N used to identify initial contact and toe-off events, in accordance with the default setting in Vicon Nexus. Gait variables were time-normalized to 100% of the gait cycle (from heel strike to subsequent heel strike) using Polygon 4 (Vicon Motion Systems Ltd., Oxford, UK). All five trials were averaged within the software prior to extracting the representative gait variables.

**Fig. 1:**
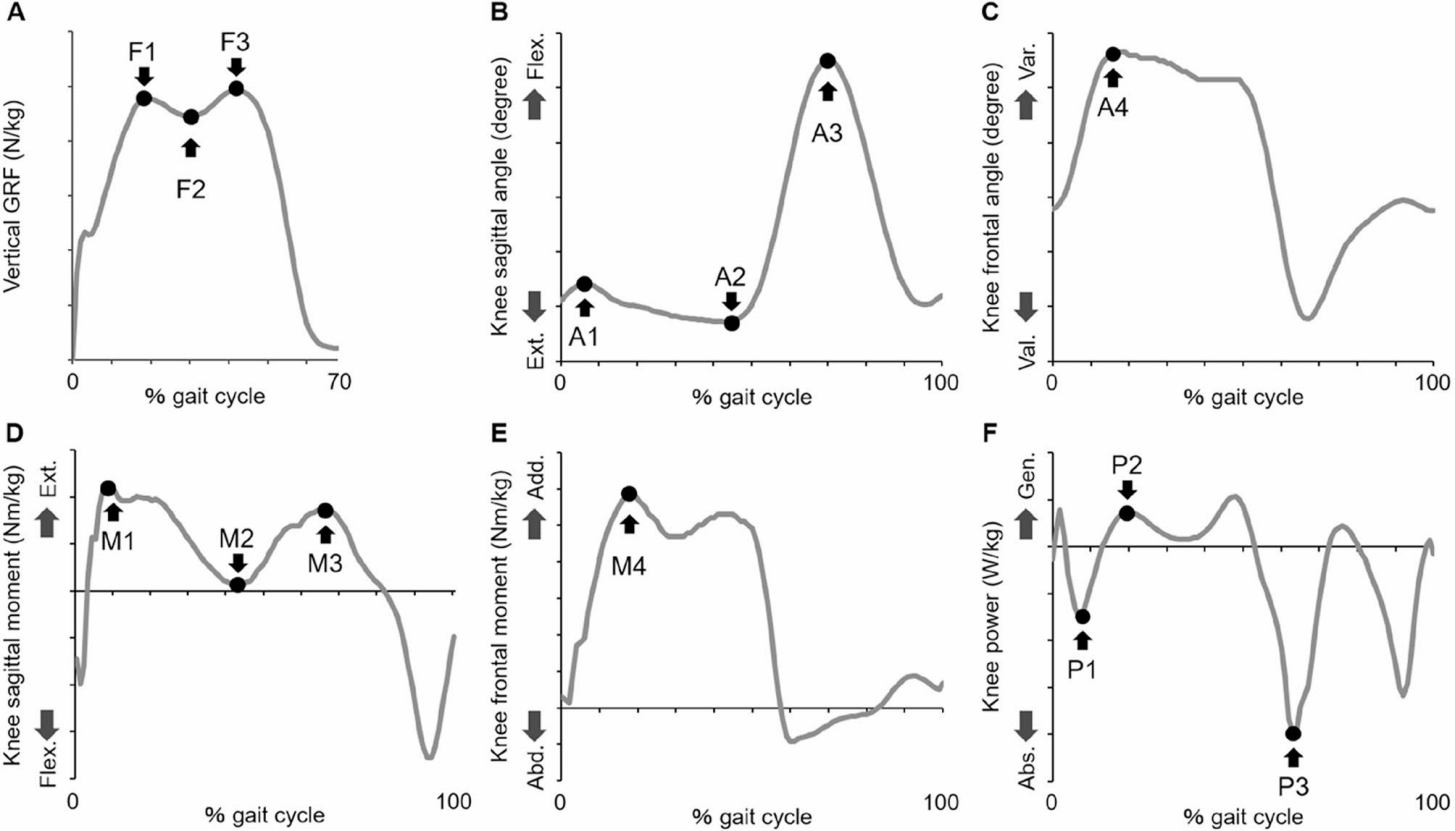
Extracted gait variables in a representative patient. Each representative extreme value in the vertical ground reaction force (**A**), knee sagittal (**B**) and frontal (**C**) angles, knee sagittal (**D**) and frontal (**E**) moments, and knee power (**F**) were extracted

The data on gait speed (m/s), step length (m), cadence (steps/min), single support time (s), and double support time (s) were extracted as temporal parameters. Other variables were extracted using their respective representative peak values, as shown in Fig. 1. Specifically, three peak values were extracted for the vertical ground reaction force (F1, F2, and F3 in Fig. 1A). In four participants, the vertical ground reaction force pattern showed a relatively shallow trough between the first and second peaks, with the minimum value (F2) slightly exceeding the first peak (F1). In these cases, a local inflection point was identified between F1 and F3, and this point was consistently used to define F2. All participants exhibited a distinguishable first and second peak, and no trials were excluded due to atypical vertical ground reaction force patterns. For the sagittal knee-joint angle during walking, the data on the maximum flexion angle in the stance phase (A1), maximum extension angle in the stance phase (A2), and maximum flexion angle in the swing phase (A3) were extracted (Fig. 1B). The frontal knee-joint angle data was extracted as the maximum varus angle during the stance phase (A4 in Fig. 1C). The data on the sagittal knee-joint moments were extracted as the maximum extension moment in the early stance phase (M1), maximum flexion moment in the mid-stance phase (M2), and maximum extension moment in the terminal stance phase (M3) (Fig. 1D). The frontal knee-joint moment was extracted as the maximum adduction moment during the stance phase (M4 in Fig. 1E). For knee-joint power, the data on the maximum absorption in the early stance phase (P1), maximum generation in the stance phase (P2), and maximum absorption in the terminal stance phase (P3) were extracted (Fig. 1F).

### Statistical analysis

Descriptive data are presented as mean ± standard deviation (SD) for continuous variables and as counts for nominal variables.

All measurements were visually checked for distribution and normality by group using Shapiro–Wilk test. First, to comprehensively examine the effects of pain catastrophizing on objective knee function and gait pattern, participants were divided into a high-PCS group (score ≥ 30) and a low-PCS group (score < 30) based on the official PCS manual, which defines a score of 30 as the threshold for clinically relevant levels of catastrophizing [22]. This cut-off has also been used in a previous clinical study [23]. For intergroup comparisons, t-test was used for normally distributed data and Mann–Whitney U test for non-normally distributed data. Fisher’s exact test was used for between-group comparisons of the nominal variables. To test our hypotheses, we used hierarchical multiple regression analysis to assess the unique contribution of pain catastrophizing on objective knee function and gait variables. The dependent variables for the hierarchical multiple regression analysis were knee-flexion ROM, quadriceps strength, gait speed, first peak of the ground reaction force (F1), and maximum knee-flexion angle in the swing phase (A3). Sex, age, BMI, and pain intensity could be potential confounders [24–27]. In each model, PCS was entered in the final step (step 3) after controlling for age and sex, which was entered in step 1, and BMI and VAS pain scores during walking were entered in step 2. Five separate hierarchical multiple regression models were constructed, each with a single dependent variable. Specifically, three gait variables—gait speed, F1, and A3—were analyzed independently. Likewise, two objective knee function variables—knee-flexion ROM and quadriceps strength—were modeled separately. These outcomes were selected based on our clinical hypothesis that patients with high pain catastrophizing would demonstrate gait patterns characterized by load avoidance and reduced knee mobility, along with diminished objective knee function. Modeling each outcome independently allowed for greater conceptual clarity and minimized the risk of multicollinearity, particularly given the modest sample size. The results of the regression analysis are expressed as standardized regression coefficients (β), *P*-values, and partial coefficients of determination (partial *R*^2^).

All statistical analyses were performed using EZR version 1.63 (Jichi Medical University Saitama Medical Center, Saitama, Japan) [28] graphical user interface for R version 4.20 (R Foundation for Statistical Computing, Vienna, Austria) and IBM SPSS Statistics for Windows, version 23.0 (IBM Corp., Armonk, N.Y., USA). Statistical significance was defined as *P* < 0.05. Because this was an exploratory study using retrospective data, an a priori sample size calculation was not performed. To assess the adequacy of the sample size for regression analyses, we performed a post hoc power analysis using the pwr.f2.test() function in the pwr package (version 1.3-0) in R (version 4.2.0; R Foundation for Statistical Computing, Vienna, Austria). Additionally, a sensitivity analysis was conducted to test the robustness of the results by excluding duplicated cases (i.e., patients who underwent bilateral knee arthroplasty). The same regression models were applied to the resulting dataset of 63 unique cases.

## Results

During the study period, 63 patients and 75 knees were analyzed. The patients were divided into a high-PCS group (52 patients and 60 knees) and a low-PCS group (13 patients and 15 knees). As PCS was evaluated for each unilateral surgery, two patients had one knee categorized into the high PCS group and the other into the low PCS group. In other words, the PCS of the two patients changed with each unilateral surgery. Among all bilateral cases, the interval between the two surgeries ranged from 2 to 14 months (mean ± SD: 7.2 ± 4.4 months). The PCS was administered separately and independently before each procedure.

###  Comparison of demographic data between high- and low-PCS groups

The demographic data for both groups are shown in Table 1. There were no significant differences between the groups in terms of age, sex, BMI, and affected side. In addition, there was no significant difference between the groups in VAS pain at rest; however, the patients in the high-PCS group had significantly higher VAS pain scores during walking than those in the low-PCS group (*P* < 0.001).

**Table 1:** Participant demographics based on PCS

	High PCS (≥ 30) *n* = 60	Low PCS ( *n* = 15	*P*-value ^a^
Age (years)	75.1 ± 5.6	73.5 ± 7.8	0.389
Sex, Female/Male (n)	43/17	11/4	1.000
Body mass index (kg/m^2^)	26.5 ± 4.0	26.6 ± 2.9	0.672
Affected side, Left/Right (n)	34/26	8/7	1.000
VAS pain score at rest (mm)	10.0 ± 18.7	4.1 ± 6.5	0.693
VAS pain score during walking (mm)	64.3 ± 24.9	39.6 ± 22.5	
PCS (pts)	43.0 ± 5.7	20.7 ± 7.1	—

*VAS* visual analogue scale, *PCS* Pain Catastrophizing Scale

^a^ Values obtained using two-tailed unpaired t-test or Mann–Whitney U test for continuous variables, and Fisher’s exact test for sex and side distribution

The values are presented as mean ± standard deviation or numbers

### Comparison of objective knee function between high- and low-PCS groups

The knee ROM and muscle strength results for both groups are shown in Table 2. There were no significant differences in flexion and extension ROM between the groups. Additionally, there were no significant differences in quadriceps and hamstring strength between the groups.

**Table 2:** Knee range of motion and muscle strength in each group based on PCS

	High PCS (≥ 30)	Low PCS (	*P*-value ^a^
Knee range of motion ^b^			
Flexion (°)	125.9 ± 15.7	125.0 ± 16.8	0.863
Extension (°)	−6.4 ± 7.0	−10.0 ± 9.1	0.230
Muscle strength ^c^			
Quadriceps (% body weight)	65.3 ± 24.6	65.1 ± 8.9	0.962
Hamstrings (% body weight)	35.6 ± 16.0	34.5 ± 9.1	0.738

*PCS* Pain Catastrophizing Scale

^a^ Values obtained using two-tailed unpaired t-test or Mann–Whitney U test for continuous variables and Fisher’s exact test for sex and side distribution

^b^ Missing data on seven patients (five with high PCS and two with low PCS)

^c^ Missing data for four patients (three with high PCS and one with low PCS)

The values are presented as mean ± standard deviation

### Comparison of gait variables between high- and low-PCS groups

The gait variables for both groups are presented in Table 3. No significant differences were found between the two groups in any of the gait variables, including the vertical ground reaction force and sagittal knee angle, which were expected to differ based on the hypothesis.

**Table 3:** Gait variables in each group based on PCS

	High PCS (≥ 30)	Low PCS (	*P*-value ^a^
Temporal parameter			
Gait speed (m/s)	0.76 ± 0.25	0.78 ± 0.17	0.825
Step length (m)	0.43 ± 0.10	0.43 ± 0.10	0.913
Cadence (steps/min)	102.65 ± 18.3	107.2 ± 15.0	0.596
Single support time (s)	0.40 ± 0.05	0.39 ± 0.06	0.688
Double support time (s)	0.39 ± 0.26	0.34 ± 0.09	0.657
Vertical ground reaction force (N/kg)			
F1	9.77 ± 0.51	9.50 ± 0.63	0.090
F2	8.94 ± 0.55	8.77 ± 0.51	0.231
F3	9.64 ± 0.39	9.54 ± 0.56	0.340
Sagittal knee angle (°)			
Maximum flexion in the stance phase (A1)	12.9 ± 12.0	16.0 ± 10.4	0.371
Maximum extension in the stance phase (A2)	8.4 ± 12.1	11.8 ± 9.3	0.337
Maximum flexion in the swing phase (A3)	41.3 ± 13.6	42.6 ± 16.9	0.525
Frontal knee angle (°)			
Maximum varus in the stance phase (A4)	9.7 ± 6.7	10.4 ± 10.4	0.804
Sagittal knee moment (Nm/kg)			
Maximum extension in the early stance phase (M1)	0.26 ± 0.18	0.29 ± 0.29	0.701
Maximum flexion in the mid-stance phase (M2)	0.00 ± 0.33	0.09 ± 0.31	0.375
Maximum extension in the terminal stance phase (M3)	0.24 ± 0.18	0.27 ± 0.19	0.716
Frontal knee moment (Nm/kg)			
Maximum adduction in the stance phase (M4)	0.69 ± 0.19	0.60 ± 0.24	0.145
Knee power (W/kg)			
Maximum absorption in the early stance phase (P1)	0.28 ± 0.30	0.28 ± 0.21	0.827
Maximum generation in the stance phase (P2)	0.18 ± 0.20	0.15 ± 0.12	0.854
Maximum absorption in the terminal stance phase (P3)	0.57 ± 0.40	0.58 ± 0.31	0.795

*PCS* Pain Catastrophizing Scale

^a^ Values obtained using two-tailed unpaired t-test or Mann–Whitney U test for continuous variables, and Fisher’s exact test for sex and side distribution. The values are presented as mean ± standard deviation

### Hierarchical multivariate linear regression analysis on the association between gait variables and PCS

The associations between gait variables and PCS scores using hierarchical multivariate linear regression analysis are shown in Table 4. In the present study, there was no association between the degree of PCS and gait speed, first peak of the vertical ground reaction force (F1), or maximum knee-flexion angle in the swing phase (A3). Only sex accounted for 7.7% of the variance in gait speed and 6.9% of the variance in the maximum knee-flexion angle in the swing phase (A3).

**Table 4:** Hierarchical multivariate linear regression analysis of the association between gait variables and PCS in all patients (75 knees)

	Dependent variables: gait variables								
	Gait speed (m/s)			F1 of the vertical GRF (N/kg)			Maximum flexion angle in the swing phase (A3; °)		
Independent variables	β	*P*	Partial *R*^2^	β	*P*	Partial *R*^2^	β	*P*	Partial *R*^2^
Step 1 Age Sex	−0.215 −0.281	0.060 0.015	0.045 0.077	0.048 0.026	0.688 0.829	0.002 0.001	−0.047 −0.265	0.681 0.024	0.002 0.069
Step 2 BMI VAS during walking	−0.102 −0.095	0.399 0.404	0.009 0.009	−0.195 −0.074	0.128 0.531	0.033 0.005	0.010 −0.028	0.936 0.809	0.000 0.001
Step 3 PCS	0.048	0.709	0.002	0.114	0.392	0.010	−0.018	0.891	0.000

*VAS* visual analogue scale, *PCS* Pain Catastrophizing Scale, *GRF* ground reaction force

Bold values indicate significant associations based on regression analysis (*P* < 0.05)

### Hierarchical multivariate linear regression analysis on the association between objective knee function and PCS

Table 5 shows the association between objective knee function and PCS based on hierarchical multivariate linear regression analysis. In the present study, no association was found between the degree of PCS score and flexion ROM or quadriceps strength. Sex accounted for 17.6% of the variation in the quadriceps strength.

**Table 5:** Hierarchical multivariate linear regression analysis of the association between objective knee function and PCS in all patients (75 knees)

	Dependent variables: objective knee variables					
	Flexion ROM (degree)			Quadriceps strength (% body weight)		
Independent variables	β	*P*	Partial *R*^2^	β	*P*	Partial *R*^2^
Step 1 Age Sex	−0.066 0.134	0.599 0.284	0.004 0.017	−0.052 −0.424	0.640 0.000	0.003 0.176
Step 2 BMI VAS during walking	−0.103 0.198	0.440 0.113	0.009 0.038	−0.069 −0.028	0.565 0.806	0.004 0.001
Step 3 PCS	−0.080	0.585	0.005	−0.069	0.595	0.004

*VAS*,visual analogue scale, *PCS* Pain Catastrophizing Scale, *ROM* range of motion, *BMI* body mass index

Bold values indicate significant associations based on regression analysis (*P* < 0.05)

A post hoc power analysis for the final hierarchical models was conducted. Assuming five predictors and a medium effect size (f² = 0.15), the estimated statistical power was approximately 0.70. This suggests a moderate capacity to detect medium-sized associations in the current sample.

In the sensitivity analysis using only one knee per patient (*n* = 63), hierarchical multiple regression analyses yielded results consistent with those of the full sample. PCS scores were not significantly associated with any of the gait or knee function variables, and their incremental explanatory value (ΔR²) remained negligible (ranging from 0.00005 to 0.006). Details of these analyses are provided in Supplementary Table 1.

## Discussion

The main finding of this study was that patients with severe knee OA showed no significant differences in objective knee function and gait variables when divided according to PCS cut-off values. Further hypothesized associations between PCS and gait speed, first peak of vertical ground reaction force, maximum knee-flexion angle in the swing phase, flexion ROM, and quadriceps strength were examined; however, no association was observed. These results did not support our hypothesis that patients with severe pain catastrophizing exhibit gait characterized by load avoidance and immobilization of the knee joint with a concomitant decline in objective knee function. In this study, the term “objective knee function” refers specifically to joint-level physical performance indicators, namely passive ROM and isokinetic quadriceps strength. These measures differ conceptually from broader definitions of functional ability commonly assessed using performance-based tests (e.g., timed up-and-go) or patient-reported outcome measures (PROMs). Caution is therefore warranted when comparing our results with studies that use alternative definitions of function.

Many previous studies have reported a relationship between pain catastrophizing and knee-joint function assessed using PROMs in patients with knee OA [14, 29–33]. Most of these studies found that higher levels of pain catastrophizing were associated with poorer subjective assessments of knee function and physical activity [14, 29–32]. However, one report did not replicate this relationship [33], and the findings remain inconsistent. Furthermore, there is a paucity of studies exploring the relationship between pain catastrophizing and objective measures of knee function, such as ROM and muscle strength. Clarifying this relationship is important for understanding the disability profile of patients with knee OA from a more comprehensive perspective. In the present study, we found no statistically significant differences in knee ROM between groups stratified by PCS cut-off values. Hierarchical multivariate regression analysis also did not identify any significant association between flexion ROM and PCS scores. These findings are in line with the results of Suzuki et al. [29], although participant characteristics and methodologies differed. Suzuki et al. [29] included a broader range of OA severity (KL grade 1–4), whereas our study included only patients with more advanced OA (KL grade ≥ 3). Similarly, no significant differences in quadriceps muscle strength were observed between PCS-based groups, and the regression analysis did not indicate a clear association between muscle strength and PCS score. While previous studies using subjective evaluations suggested poorer function in patients with high PCS scores, studies using performance-based metrics have shown mixed results. For instance, one prior study found no significant relationship between isokinetic quadriceps strength and pain catastrophizing in female patients with early-to-severe knee OA [34], supporting our findings. The discrepancy between subjective and objective assessments may be explained by previous reports demonstrating poor concurrent validity between PROMs and performance-based assessments in patients with knee OA and those undergoing total knee arthroplasty [35, 36]. These inconsistencies suggest that pain catastrophizing may influence patients’ perceptions more than their measurable physical capabilities. Therefore, when assessing disability in patients with severe knee OA, it is important to consider that pain catastrophizing may not be directly associated with objectively measured knee function.

In a previous study [16], several gait variables—similar to those assessed in the present study—were evaluated before surgery in a comparable patient population, and some effects of pain catastrophizing were observed. Interestingly, a more favorable knee-joint extension angle during mid-stance was observed in patients with higher PCS scores. It is unclear whether these results correspond to the kinematic features of the fear-avoidance model [37], which proposes that catastrophic interpretation of pain causes pain-related fear, which in turn drives behaviors such as hypervigilance, avoiding, and/or escaping activities expected to reproduce pain and/or injury. The present study did not replicate the findings of this previous study [16], and our results suggest that the association between pain catastrophizing and gait patterns in patients with severe knee OA may be limited. One possible explanation for this discrepancy is that although both studies evaluated similar gait variables in severe knee OA patients (KL grade ≥ 3), there were important differences in methodology. Our study used a higher PCS cut-off score (≥ 30) to identify clinically relevant levels of catastrophizing, whereas Harato et al. [16] used a lower threshold of 23 points. Additionally, our analyses adjusted for confounding factors such as BMI and walking pain using multivariate regression, whereas Harato et al. [16] performed unadjusted group comparisons. Differences in sample size and participant age may also contribute, as our cohort had a higher mean age and a predominance of females. Taken together, these differences may explain the contrasting findings. Given the limitations of the present study, it remains unclear whether pain catastrophizing meaningfully affects gait biomechanics in patients with severe knee OA; however, our findings do not support a strong association.

However, some studies have provided evidence of an association between pain catastrophizing and changes in neuromotor behavior during gait [14, 38, 39]. Pakzad et al. [38] examined the relationship between pain catastrophizing and trunk muscle activity during gait in participants with non-specific low back pain. They reported that participants with higher pain catastrophizing had higher electromyography activation of specific trunk muscles and less phasic modulation of trunk muscles during gait, suggesting that these changes are non-functional and maladaptive behaviors. Van Wyngaarden et al. [39] also showed that greater pain catastrophizing at 6 weeks after lower extremity fracture was associated with a lower loading rate, longer stance time, higher impulse on the injured limb, and lower 6-min walk test distance at 12 months. The difference between these studies [14, 38, 39] and the present study was the participant population. The patients with knee OA may have altered gait patterns depending on the severity of the disease [40] and their association with symptoms [41]. Patients with severe knee OA, the participants of this study, may not be capable of adapting loading strategies that reflect pain catastrophizing in knee-joint biomechanics during gait. The results of the present study suggest that pain catastrophizing may not affect the knee joint biomechanics during gait in patients with severe knee OA. Therefore, pain catastrophizing and knee biomechanics during gait should be evaluated and treated independently to understand the disability status of patients with severe knee OA.

The present study has some limitations. First, the gait variables examined in this study were limited to representative peak values for the knee joint. Thus, its influence on the other lower extremity joints is unknown. Second, multiple patients with left and right knee joints with OA were included at different times. The condition of the contralateral knee joint varied with each case because the current study evaluated the data before surgery. Although we cannot deny that this may have affected the results, we considered the condition of the participants as encountered in clinical practice. Third, as the study was limited to patients with severe knee OA for whom surgery was planned, caution must be exercised when generalizing the results. Fourth, although the post hoc power analysis indicated a moderate level of statistical power to detect medium-sized effects, it should be noted that the incremental effect of PCS (ΔR²) in the final model was small. Therefore, the possibility of a Type II error cannot be excluded, particularly regarding subtle associations. Larger-scale studies with a priori power calculations are warranted to further explore these relationships. Fifth, although some patients were included twice due to bilateral involvement, PCS scores in these cases showed intra-individual variation over time, likely reflecting the situational nature of pain catastrophizing. This interpretation is suggested by previous research demonstrating day-to-day variability in PCS scores in response to pain intensity [42]. Nonetheless, we conducted a sensitivity analysis excluding these cases and indicated that our main conclusions remained unchanged. In these bilateral cases, the interval between surgeries ranged from 2 to 14 months, which may have influenced patients’ psychological state at the time of the second assessment. Despite these limitations, the present results will help understand the relationship between pain catastrophizing, objective knee function, and knee biomechanics during gait in patients with severe knee OA.

## Conclusion

In conclusion, the results indicate that no significant association was observed between pain catastrophizing and objective knee function or knee biomechanics during gait in patients with severe knee OA. Therefore, objective knee function, knee biomechanics during gait, and pain catastrophizing may need to be independently assessed and treated to better understand the disability status of patients with severe knee OA.

## Supplementary Information

Supplementary Material 1.

## Data Availability

The datasets generated during and/or analyzed during the present study are available from the corresponding author on reasonable request.

## Abbreviations

OA: Osteoarthritis
PCS: Pain Catastrophizing Scale
ROM: Range of Motion
BMI: Body Mass Index
VAS: Visual Analog Scale
SD: Standard Deviation
GRF: Ground Reaction Force
PROMs: Patient-reported outcome measures
