# Supplementary material for: Association of knee joint performance and gait patterns with pain catastrophizing in patients with severe knee osteoarthritis: a cross-sectional study
Source: BMC Musculoskelet Disord. 2025 Jul 28;26:724. doi: 10.1186/s12891-025-08993-2 (PMC12306094; doi:10.1186/s12891-025-08993-2)
Supplement: Supplementary file 1 — Supplementary Material 1. [file 12891_2025_8993_MOESM1_ESM.docx]

**Supplementary table 1.** Sensitivity analysis excluding bilateral cases (n = 63)

| Dependent Variables | R² (Full Model) | Adjusted R² | PCS Coefficient (β) | PCS p-value | ΔR² (PCS Contribution) | Model p-value |
| --- | --- | --- | --- | --- | --- | --- |
| Gait Speed (m/s) | 0.126 | 0.050 | 0.0017 | 0.595 | 0.006 | 0.162 |
| F1 of the vertical GRF (N/kg) | 0.083 | 0.002 | 0.0014 | 0.851 | 0.0006 | 0.411 |
| Maximum flexion angle in the swing phase (A3; °) | 0.063 | -0.019 | 0.0106 | 0.956 | 0.00005 | 0.575 |
| Flexion ROM (degree) | 0.061 | -0.031 | -0.070 | 0.780 | 0.0014 | 0.649 |
| Quadriceps strength (% body weight) | 0.190 | 0.113 | -0.099 | 0.745 | 0.002 | 0.043 |

Each model included sex and age in Step 1, BMI and walking pain (VAS) in Step 2, and PCS scores in Step 3. ΔR² indicates the increase in explanatory power after PCS was added to the model in Step 3.

*BMI,* body mass index, *VAS*, visual analogue scale, *PCS*, Pain Catastrophizing Scale
